# The effects of early exercise on brain damage and recovery after focal cerebral infarction in rats

**DOI:** 10.1111/j.1748-1716.2010.02174.x

**Published:** 2011-02

**Authors:** F Matsuda, H Sakakima, Y Yoshida

**Affiliations:** School of Health Sciences, Faculty of Medicine, Kagoshima UniversityKagoshima, Japan

**Keywords:** angiogenesis, midkine, nerve growth factor, neurogenesis, rehabilitation

## Abstract

**Aim:**

Exercise can be used to enhance neuroplasticity and facilitate motor recovery after a stroke in rats. We investigated whether treadmill running could reduce brain damage and enhance the expression of midkine (MK) and nerve growth factor (NGF), increase angiogenesis and decrease the expression of caspase-3.

**Methods:**

Seventy-seven Wistar rats were split into three experimental groups (ischaemia-control: 36, ischaemia-exercise: 36, sham-exercise: 5). Stroke was induced by 90-min left middle cerebral artery occlusion using an intraluminal filament. Beginning on the following day, the rats were made to run on a treadmill for 20 min once a day for a maximum of 28 consecutive days. Functional recovery after ischaemia was assessed using the beam-walking test and a neurological evaluation scale in all rats. Infarct volume, and the expression of MK, NGF, anti-platelet-endothelial cell adhesion molecule (PECAM-1), and caspase-3 were evaluated at 1, 3, 5, 7, 14 and 28 days after the induction of ischaemia.

**Results:**

Over time motor coordination and neurological deficits improved more in the exercised group than in the non-exercised group. The infarct volume in the exercised group (12.4 ± 0.8%) subjected to treadmill running for 28 days was significantly decreased compared with that in the control group (19.8 ± 4.2%, *P* < 0.01). The cellular expression levels of MK, NGF and PECAM-1 were significantly increased while that of caspase-3 was decreased in the peri-infarct area of the exercised rats.

**Conclusions:**

Our findings show that treadmill exercise improves motor behaviour and reduces neurological deficits and infarct volume, suggesting that it may aid recovery from central nervous system injury.

Stroke is a leading cause of serious long-term physical disability. There is increasing evidence that physical activity is associated with decreased incidence of stroke in humans (Evenson *et al.* 1999, Hu *et al.* 2000, Greenlund *et al.* 2002, Lee *et al.* 2003a). Physical exercise may ameliorate neurological impairment by impeding neuronal loss following various brain insults, and exercise has now been proposed as a possible means of treatment for such conditions, and especially in stroke patients. Exercise has beneficial effects on brain function, including the promotion of plasticity. Clinical data strongly favour early mobilization and training (Johansson 2000). Several studies have substantiated the beneficial effects of early exercise on ischaemia-induced brain injury in animal models (Stummer *et al.* 1994, 1995, Wang *et al.* 2001, Ang *et al.* 2003, Endres *et al.* 2003). Although the behavioural improvements and structural alterations in the brain that occur following injury to it are well documented and may be associated with neurotrophic factors, little is known regarding the mechanisms underlying them.

Neurotrophic factors play roles in neuronal survival, proliferation, maturation and outgrowth in the developing brain and also have neuroprotective functions after insult in the mature brain. Midkine (MK) has been implicated in the repair of several tissues, as it is expressed during the early stages of experimental cerebral infarction (Yoshida *et al.* 1995), peripheral nerve injury (Sakakima *et al.* 2004a,b;), spinal cord injury (Sakakima *et al.* 2004a,b;), bone fracture (Ohta *et al.* 1999), myocardial infarction (Obama *et al.* 1998) and skin burns (Iwashita *et al.* 1999). MK has *in vivo* angiogenic (Choudhuri *et al.* 1997) and neuronal survival-promoting activities (Owada *et al.* 1999). Although the neurotrophic activities of MK suggest that the increased levels of it that follow brain injury may have protective and/or regenerative effects, it is unknown whether MK expression is increased by exercise in the brain after focal brain ischaemia and whether overexpression of MK is associated with the reduction of brain lesions, promotion of angiogenesis, and/or decreased apoptotic activity in brain lesions.

The members of the neurotrophin family, including nerve growth factor (NGF) and brain-derived neurotrophic factor (BDNF), are of particular interest with regard to the promotion of cell growth and enhancement of neuronal activity (Gomez *et al.* 1997, Ickes *et al.* 2000). Physical exercise has been shown to increase the production of the mRNA of neurotrophins such as NGF and BDNF in rat brain (Neeper *et al.* 1996). Environmental enrichment leads to regional increases in NGF, BDNF and neurotrophin-3 levels in the rat brain (Torasdotter *et al.* 1998, Ickes *et al.* 2000). In rat brains, angiogenesis has been reported in response to motor exercise (Black *et al.* 1990, Isaacs *et al.* 1992, Kleim *et al.* 2002, Swain *et al.* 2003). Although recent studies have reported associations between neurotrophic factors and motor recovery after cerebral ischaemia, these studies used different models of stroke in rats and examined only one neurotrophic factor or one time point. It remains unclear whether the endogenous production of neurotrophins and angiogenesis induced by physical exercise have neuroprotective effects after stroke.

In the present study, we examined rats after transient middle cerebral artery (MCA) occlusion to determine whether (1) regular motor exercise on a treadmill immediately after ischaemia/reperfusion injury reduced neurological deficits and infarct volume; (2) the neuroprotective effect of exercise was associated with cellular expression of MK and NGF in the cortex and striatum as well as angiogenesis in regions of the brain supplied by the MCA and (3) effects of treadmill exercise on ischaemia/reperfusion-induced neuronal injury in rats are recognizable.

## Material and methods

### Animals

Seventy-nine male Wistar rats (Japan SLC, Tokyo, Japan) weighing 220–260 g (approx. 8 weeks of age) at the time of surgery were used in this study. The rats were randomly assigned to the ischaemia-non-exercised control (IC; *n* = total 37; *n* = 6 at 1-, 3-, 5-, 7-, 14-, and 28-day time points; one rat expired within 24 h of surgery), sham-exercise (SE; *n* = 5) and ischaemia-exercise (IE; *n* = total 37; *n* = 6 at 1-, 3-, 5-, 7-, 14-, and 28-day time points; one rat expired within 24 h of surgery) groups before surgery. The SE rats underwent sham occlusion, the IC and IE rats underwent left MCA occlusion, and the SE and IE rats ran on a treadmill. Two rats died within 24 h after insult because of severe cerebral infarction, possibly because of atypical MCA variations. One of the rats that died belonged to the IE group, and the other was from the IC group. These rats were excluded from the study. No rat died between 24 h after the stroke and end of the experiments as a result of treadmill exercise or disease. Seventy-seven rats completed the experiment. The animals were socially housed (2–3 rats per cage) under a 12 h reverse light/dark cycle in clear Plexiglas cages with water and food available *ad libitum*. The experimental protocols accorded with established guidelines determined by the Animal Care Committee of the Ethics Board of the Institute of Laboratory Animal Sciences of Kagoshima University.

The animals were anaesthetized via the injection of 4% chloral hydrate (10 mL kg^−1^, intraperitoneally) and were given further doses as necessary to ensure adequate anaesthesia during surgery. Rectal temperature was monitored throughout the surgical procedures and was maintained at 37 °C using a heating blanket controlled by an electronic temperature controller (KN-474; NATUME, Tokyo, Japan). Stroke was induced by 90 min left MCA occlusion using an intraluminal filament (Longa *et al.* 1989). Briefly, a midline incision was made, and the left common carotid artery (CCA), the external carotid artery (ECA) and the internal carotid artery (ICA) were exposed. The distal ECA branch was coagulated completely. The CCA and ECA were then tied with a white thread. A monofilament (a 4-0 nylon suture with a blunted tip of 0.2–0.3 mm in diameter and 16 mm in length) was inserted into the left CCA via an arteriotomy. It was then passed up the lumen of the ICA into the intracranial circulation and lodged into the narrow proximal anterior cerebral artery, blocking the MCA at its origin. After 90 min of MCA occlusion, reperfusion was established by withdrawal of the filament. Regional cerebral blood flow was measured by a laser Doppler flowmeter (ALF-21; Advance Co, Tokyo, Japan) before and during MCAO. A probe in the shape of a flat rectangular sheet (7.5, 3.5 and 1.0 mm in length, width and thickness respectively) was placed over the ischaemic side in the natural pocket formed between the temporal muscle and the lateral aspect of the skull. All animals exhibited significantly reduced blood flow (a >20% reduction compared with the pre-ischaemic baseline) during MCA occlusion, and none were excluded from the study because of lack of reduction of blood flow. For sham occlusion, the left CCA was exposed following general anaesthesia similar to the procedure for stroke induction. The ICA was separated at the junction of the left ICA and the left ECA but was not occluded. Body temperature was maintained as described above.

### Exercise training

The rats in both the SE and IE groups underwent treadmill running, which began 24 h after the surgery. The rats in the IC group were allowed to move freely in their cages, but no additional treadmill running was employed. The rats in the IE group underwent treadmill running for 20 min a day every day for a maximum of 28 days. The rats in the SE group ran on a treadmill for 28 consecutive days after surgery. A motorized treadmill (Rat runner, RR-1200; AKK, Shimane, Japan) that forced the rats to run via an electric stimulation system installed on the rear floor was used. All animals ran at a speed of 3 m min^−1^ for the first 3 days after surgery, 8 m min^−1^ from 4 to 6 days after surgery and then 13 m min^−1^ on subsequent days with an inclination of 0°. Two animals were housed per cage, and all animals were allowed to recover in their cages in a warm environment with food and water supplied *ad libitum*. The exercises were performed at normal room temperature, in a normal light setting and with normal room noise. To monitor the stress induced in the rats by treadmill running, body weight was measured periodically (Ding *et al.* 2004b). None of the animals were excluded from the study because of an unwillingness to run.

### Motor behaviour and neurological assessments

Animals from the SE, IE and IC groups were examined with a motor test paradigm (limb placing: motor behaviour test) and for neurological deficits before and 1, 3, 5, 7, 14 and 28 days after surgery. All rats were tested three times on each trial day.

In the motor behaviour test, which was modified from the scoring system of Fenny *et al.* (1982), the locomotion of the rats was evaluated pre-operatively and post-operatively using a beam-walking task with an elevated narrow beam (150 cm long × 2.5 cm wide). The worst score (‘0’) was given if the rat was unable to traverse the beam and could neither place the affected limbs on the horizontal surface nor maintain balance. A score of ‘1’ was given if the rat was unable to traverse the beam or to place the affected limbs on the horizontal surface of the beam but was able to maintain balance. A score of ‘2’ was given if the rat was unable to traverse the beam but placed the affected limbs on the horizontal surface of the beam and maintained balance. A score of ‘3’ was given if the rat used the affected limbs in less than half of its steps along the beam. A score of ‘4’ was given if the rat traversed the beam and used the affected limbs to aid more than 50% of its steps along the beam. A score of ‘5’ was given if the rat traversed the beam normally with no more than two foot slips. The day prior to surgery, the animals in all groups underwent the motor behaviour test to ensure that their performance score was 5.

A neurological grading system with a 5-point scale (0–4) described by Menzies *et al.* (1992) was used: 0 = no apparent deficits; 1 = right forelimb flexion; 2 = decreased grip of the right forelimb while tail pulled; 3 = spontaneous movement in all directions while right circling only if pulled by tail; 4 = spontaneous right circling.

Of the two observers rating motor behaviour and performing the neurological assessments, one was not informed which rats belonged to the SE, IC, and IE groups, and his scores were mainly employed for the subsequent analyses.

### Infarct assessment

The rats in the IC and IE groups were randomly killed at 1, 3, 5, 7, 14 and 28 days (*n* = 6 at each time point) after the induction of ischaemia. The rats in the SE group were killed at 28 days after surgery.

All rats were deeply anaesthetized via an injection of 4% chloral hydrate (10 mL kg^−1^, intraperitoneally) before being killed by decapitation. The brain was carefully removed and cut into six 2-mm thick coronal sections from its frontal tip using a brain slicer. The fresh brain slices were then immersed in a 2% solution of 2,3,5-triphenyltetrazolium chloride (TTC) in physiological saline at 37 °C for 5 min and were then fixed in 4% paraformaldehyde in 0.1 m phosphate buffer (pH 7.4) at 4 °C overnight, dehydrated, embedded in paraffin, and subjected to histological and immunohistochemical analyses.

The TTC-stained sections were used to determine the infarct volume in ischaemic rats. Non-infarcted ipsilateral areas and the areas contralateral to the occluded side were measured using Scion Image software beta 4.0.3 (Scion Corp., Frederick, MD, USA). The total infarct area was multiplied by the thickness of the brain sections to obtain infarct volume. To minimize the error introduced by oedema and liquefaction after infarction, an indirect method for calculating infarct volume was used (Swanson *et al.* 1989). The non-infarcted area in the ipsilateral hemisphere was subtracted from that in the contralateral hemisphere, and infarct volume was calculated using the following formula: corrected percentage of infarct volume = (contralateral hemispheric volume−ipsilateral non-infarcted volume)/contralateral hemispheric volume.

### Histological analyses

Coronal sections (5-μm-thick) were stained with haematoxylin and eosin (HE) for histological evaluation after MCA occlusion. Immunohistochemistry was performed by the indirect immunoperoxidase method. After deparaffinization and hydration, endogenous peroxidase was blocked with methanol containing 0.9% hydrogen peroxide for 10 min. After three rinses (10 min each) with 50 mm phosphate buffered saline (PBS, pH 7.6), the sections were blocked with 10% skimmed milk for 20 min at room temperature before being individually incubated at 4 °C overnight with affinity-purified rabbit anti-MK antibody (Yoshida *et al.* 1995), rabbit anti-NGF antibody (1 : 300; Alomone, Jerusalem, Israel), and rabbit anti-caspase-3 antibody (1 : 200; Santa Cruz, Santa Cruz, CA, USA) for the evaluation of apoptosis and goat anti-platelet-endothelial cell adhesion molecule (PECAM-1) antibody (1 : 100; Santa Cruz) for the evaluation of angiogenesis. After three more rinses (10 min each) with PBS, the sections were reacted with goat anti-rabbit IgG conjugated to peroxidase-labelled dextran polymer (EnVision; Dako, Carpinteria, CA, USA) for 60 min. After rinsing the sections with PBS, immunoreactivity was visualized with diaminobenzidine/peroxide. In addition, the negative controls were rigorously examined to confirm that the observed MK and caspase-3 immunoreactivity were not the result of non-specific immunostaining.

Sections of rat brain tissues were double-immunostained as described before (Sakakima *et al.* 2004a). The slides were then incubated with rabbit anti-NGF polyclonal antibody (1 : 300; Alomone) and mouse anti-glial fibrillary acidic protein (GFAP) monoclonal antibody (1 : 500; Chemicon, Billerica, MA, USA) or mouse anti-MAP-2 monoclonal antibody (1 : 200; Chemicon) overnight at 4 °C. After being washed with PBS, the slides were incubated with Alexa Fluor 568-labelled anti-rabbit IgG (Santa Cruz) and Alexa Fluor 488-labelled anti-mouse IgG diluted 1 : 200 in PBS for 1 h. After the slides were extensively washed with PBS, mounted with 90% glycerol, and examined under an Axioskope microscope (Carl Zeiss, Oberkochen, Germany).

### Quantitative analysis of immunolabelled areas

**Figure 1:**
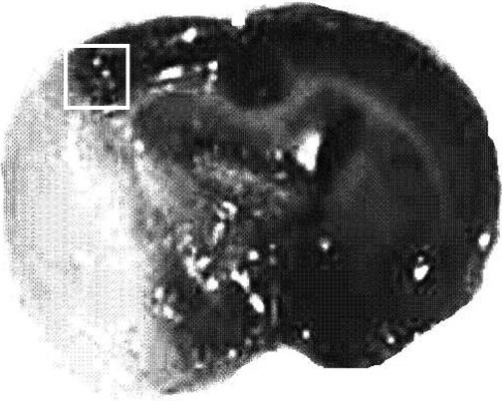
Scheme of the frontal section of the rat brain. The white-area indicates the infarcted area. The square area indicates the area examined for MK-, NGF- and PECAM-1-positive areas and caspase-3-positive cells.

The areas of immunoreactive cells in representative sections were measured in the motor cortex on the ischaemic side (Fig. 1). The same procedure was performed in the SE group. The areas of MK, NGF, PECAM-1 and caspase-3-positive cells were assessed quantitatively in three coronal sections (the sections began at intervals of 200 μm starting from the point 2 mm posterior to bregma) (2 mm × 2 mm in each field) within the frontoparietal cortex around the lesion. The areas of MK-, PECAM-1- and caspase-3-labelled cells were determined with a computer-assisted image analyzer using Scion Image software beta 4.0.3 (Scion Corp.). The number of NGF-immunoreactive cells was counted with a computer-assisted image analyzer using Adobe® Photoshop® 5.0 (Adobe Systems, San Jose, CA, USA). All histological analyses were performed in a blind fashion.

### Statistics

Motor behaviour and neurological scores were expressed as median with quartiles. Values for other variables are presented as means and standard deviations. Statistical analyses were performed using statview version 5.0 software (StatView; SAS Institute, Cary, NC, USA). Motor behaviour was evaluated, and neurological assessments were performed using the score for each trial. Two-way factorial anova analyses for different groups (IE, IC and SE groups) were used on training days (before, 1, 3, 5, 7, 14 and 28 days) to assess motor behaviour, neurological deficits, body weight, infarct volume, and areas of immunolabelling for MK, NGF, caspase-3, and PECAM-1, followed by one-way anova and *post hoc* comparisons (with Dunnett’s test) when required. Statistical significance was accepted at the level of *P*<0.05.

## Results

### Motor behaviour and neurological assessments

**Figure 2:**
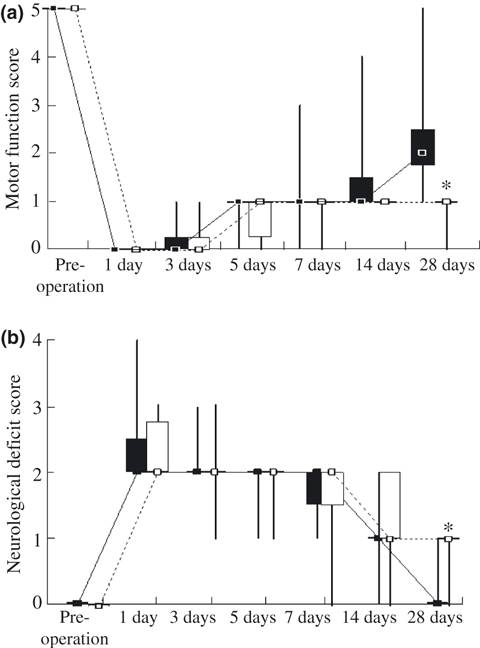
Mean motor behaviour and neurological scores of the rats after surgery. Changes in the motor function (a) and neurological (b) scores indicate the outcome of the ischaemic animals in the ischaemia-exercise (IE) (▪), ischaemia-non-exercised control (IC) (□). The motor behaviour and neurological score for the rats of the SE group were 5 and 0, respectively throughout the 28-day period of post-operative examination. The motor function score decreased after ischaemia, but improved over time. The neurological score increased after ischaemia, but gradually decreased to its value before ischaemia. Statistical analysis revealed a significant difference in motor function and neurological deficit between the two groups after 28 days. Values are shown as median with quartiles. Small box showed median value, and large box showed the 1st and the 3rd quartiles. *n* = 6 at each time point at 1, 3, 5, 7, 14 and 28 days in IE and IC groups. *n* = 5 in sham-exercise (SE) group. *n* = 36 at pre-operation in IE and IC groups. *P < 0.05 (compared with control groups).

Changes in motor behaviour and the results of neurological assessments are shown in Figure 2. In the evaluation of motor behaviour, the score of all animals before surgery was 5 (Fig. 2a). The rats of the IE and IC groups exhibited uniform, severe motor impairment at 1 day after the induction of ischaemia, as evaluated by beam-walking ability. Functional recovery was recorded at 1, 3, 5, 7, 14 and 28 days after the surgery. The rats of the IE group exhibited continuous functional recovery in the beam-walking test during the 28 days of the study. The score for the rats of the SE group was 5 throughout the 28-day period of post-operative examination, while that in the IC group was 0–1 for the first 7 post-operative days and 1–2 for the remaining 28 days. The improvement in motor behavioural score in the IE group occurred earlier than that in the IC group from 7 days after the induction of ischaemia. Two-factor factorial anova revealed significant effects of day (*F*_5,65_ = 15.125, *P*<0.0001), group (*F*_2,65_ = 565.875, *P*<0.0001) and the interaction between day and group (*F*_10,65_ = 5.452, *P*<0.0001). Subsequent one-way anova (day) revealed a significant difference among groups at 28 days (*F*_2,14_ = 31.969, *P*<0.0001) but not at other time points. In particular, at 28 days after ischaemia, Dunnett’s *post hoc* test revealed a significant difference between the IE and IC groups (*P*<0.05).

On neurological assessment, the scores for all rats before surgery were 0 (Fig. 2b). After MCA occlusion, the rats were observed for neurological deficits, and their neurological scores were found to have increased. In both the IC and IE groups, their neurological deficits improved, and their neurological score approached 0 over time. The improvement in neurological score in the IE group occurred earlier than that in the IC group. Two-factor factorial anova revealed significant effects of day (*F*_5,65_ = 17.750, *P*<0.0001), group (*F*_2,65_ = 140.536, *P*<0.0001) and the interaction between day and group (*F*_10,65_ = 5.054, *P*<0.0001). A subsequent one-way anova (day) revealed a significant difference among the groups at 28 days (*F*_2,14_ = 3.73, *P*<0.001) but not at other time points (Fig. 2b). In particular, the score in the IE group was significantly improved compared with that in the IC group at 28 days after surgery (*P*<0.01).

The body weights of the animals in the three groups gradually increased after the induction of ischaemia, without significant differences among the groups (1 day after: IE, 206.4 ± 29.5 g, IC, 222.4 ± 31.9 g, 3 days: IE, 187.9 ± 21.5 g, IC, 204.1 ± 34.1 g, 5 days: IE, 185.0 ± 25.6 g, IC, 202.0 ± 36.8 g, 7 days: IE, 208.1 ± 27.5 g, IC, 208.4 ± 40.8 g, 14 days: IE, 251.3 ± 35.7 g, IC, 245.5 ± 35.8 g, 28 days: IE, 303.3 ± 38.0 g, IC, 314.3 ± 41.7 g), suggesting that the animals did not suffer stress during exercise.

### Infarct volume

**Figure 3:**
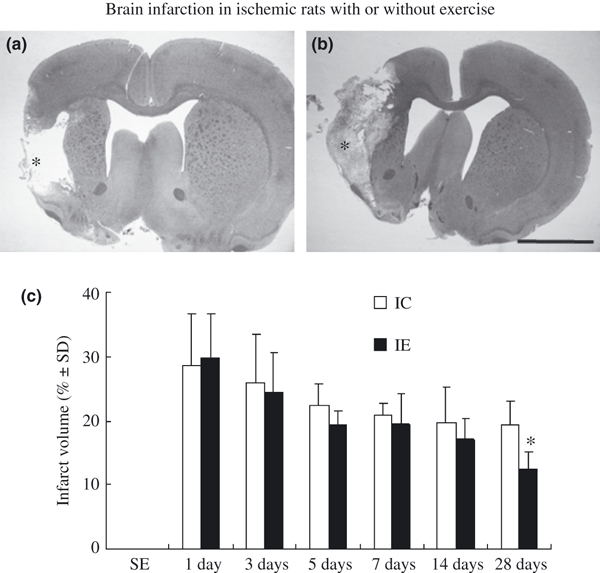
Representative photographs of infarctions after 28 days. Haematoxylin & eosin stained brains of ischaemia-exercise (IE) (a) and ischaemia-non-exercised control (IC) (b) groups after 28 days is shown. The brain infarcts were mainly observed in the cerebral cortex including the dorsolateral and lateral cortices and lateral striatum. The section shown is seen from the frontal tip. The damaged cerebral tissue underwent liquefaction and is missing in both groups (asterisk). Regarding the changes in the infarct volume of rats with or without treadmill exercise after ischaemia (c), in the rats that were subjected to forced running for 28 days, the mean infarct volume was significantly smaller than that in the controls. Values are shown as mean ± standard deviations (SD). *P < 0.05 (compared with control groups, *n* = 6).

Histological analysis with TTC staining was performed to determine the extent of brain infarction at 1, 3, 5, 7, 14 and 28 days after ischaemia. Areas of damage were observed by HE staining, mainly in the cerebral cortex including the dorsolateral and lateral cortices, as well as in the lateral striatum (Fig. 3a,b). Although our analyses failed to detect any interaction between group and day (*F*_5,60_ = 0.753, *P*=0.58), a subsequent one-way anova (day) revealed a significant difference among groups in infarct volume at 28 days (*F*_1,10_ = 4.60, *P*=0.002) but not at other time points (data not shown). At 28 days, the infarct volume in the IE group (12.4 ± 0.8%) was significantly decreased compared with that in the IC group (19.8 ± 4.2%) (Fig. 3c). This 38% reduction was highly significant (*P*<0.01). The sham-operated rats exhibited no infarction (data not shown).

### Overexpression of neurotrophic factors and angiogenesis after exercise

**Figure 4:**
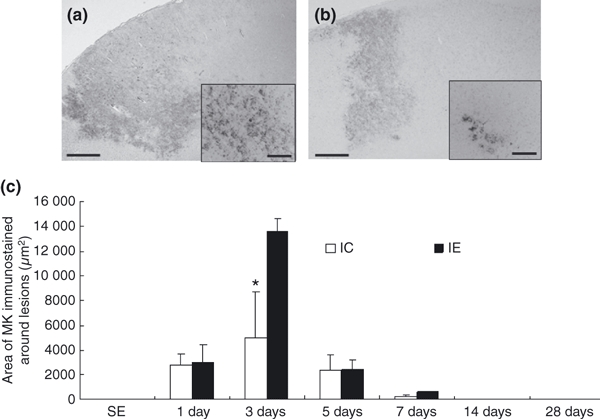
Representative photographs of midkine (MK)-positive cells (a and b) and changes in MK immunoreactivity on the infarction side after ischaemia (c). Brain sections were immunohistochemically stained for MK in the ipsilateral frontoparietal cortex including the motor cortex 3 days after ischaemic injury in the ischaemia-exercise (IE) (a) and ischaemia-non-exercised control (IC) groups (b). The rats of the IE group showed intense staining for MK immunoreactivity. Quantitative analyses were conducted on the MK immunoreactivities of the IE and IC groups in the motor cortex for 28 days. anova analysis demonstrated a significant increase in the MK-positive area after exercise; *P < 0.05 (compared with IC group value). MK was not expressed in the normal brain (SE group). Values shown are mean ± SD (*n* = 6). Scale bar = 500 μm (large windows) and 50 μm (small windows).

To elucidate the endogenous neuroprotective effects of motor exercise after stroke, brain tissues from ischaemic rats in the IE and IC groups subjected to a maximum of 28 days of treadmill exercise were processed by immunocytochemistry to assess the cellular expression of neurotrophic factors and angiogenesis in the territory supplied by the MCA. One to five days after stroke, MK immunoreactivity was detected in the peri-infarct region in the acute stage in the rats of the IE and IC groups, but was not detected on the contralateral side or in the brains of the rats of the SE group. Three days after ischaemia, MK immunoreactivity that was significantly higher in intensity in the IE group than in the IC group was observed in the peri-infarct region (Fig. 4a,b). At 14–28 days, no MK signal was detected in the ipsilateral or contralateral cortex. Two-factor factorial anova revealed significant effects of day (*F*_5,60_ = 32.43, *P*<0.0001), group (*F*_1,60_ = 11.61, *P*=0.003), and the interaction between day and group (*F*_10,60_ = 10.4, *P*=0.0001). A subsequent one-way anova (day) revealed a significant difference among groups in MK expression at 3 days (*F*_1,11_ = 7.71, *P*=0.018) but not at other time points (data not shown). In particular, at 3 days after ischaemia, Dunnett’s *post hoc* test revealed a significant difference between the IE and IC groups with regard to MK expression (*P*<0.05) (Fig. 4c).

**Figure 5:**
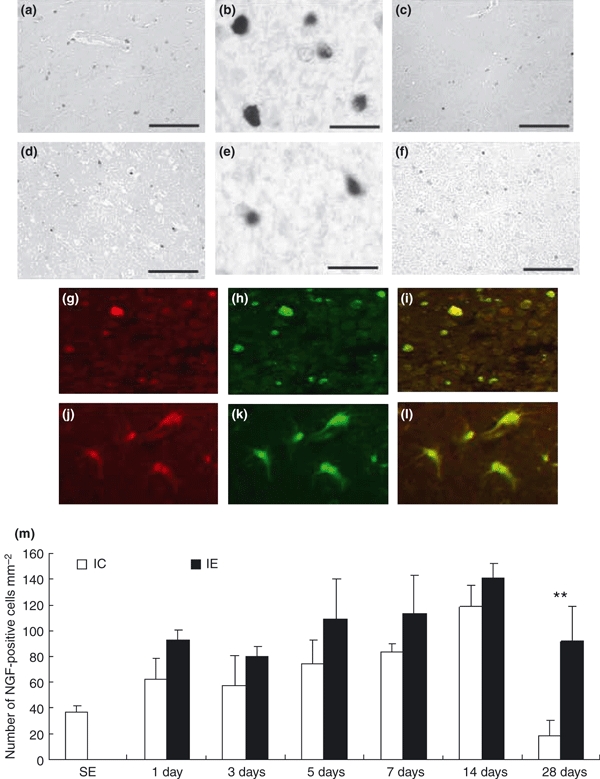
Representative photographs of nerve growth factor (NGF)-positive cells. Brain sections were immunohistochemically stained for NGF in the ipsilateral (a, b, d and e) and contralateral (c and f) frontoparietal cortices including the motor cortex 28 days after ischaemic injury in the ischaemia-exercise (IE) (a–c) and ischaemia-non-exercised control (IC) groups (d–f). Double immuno-fluorescence staining with NGF (g and j, red) and MAP-2 (h, green) or GFAP (k, green) was shown in the brain sections obtained 28 days after reperfusion in the IE group. Double staining showed that the NGF-immunoreactive cells were consistent with the neurons and astrocytes (i and l, merge). Quantitative analyses were conducted on the NGF (m) immunoreactivities of the IE and IC groups in the motor cortex of the infarct side for 28 days after ischaemia. anova analysis demonstrated a significant increase in the number of NGF-labelled cells after exercise; **P < 0.01 (compared with control value). Values shown are mean ± SD (*n* = 6). Scale bar = 30 μm (b and e) and 50 μm (a, c, d and f).

Nerve growth factor-immunopositive cells were consistently detected in the ipsilateral (Fig. 5a,b,d,e) and contralateral (Fig. 5c,f) cerebral hemispheres in the IE (Fig. 5a–c) and the IC (Fig. 5d–f) groups at 28 days after ischaemia. In particular, NGF-immunopositive cells were markedly increased in number over a widespread region around the infarct on the ipsilateral side (Fig. 5a,d). At 28 days, the number of NGF-immunoreactive neurons in the cortex in the IE group had increased more than that in the IC group (Fig. 5a). Double immunostaining with anti-NGF (Fig. 5g,j, red) and anti-MAP2 (Fig. 5h, green), as neuronal markers, or anti-GFAP (Fig. 5k, green), a marker of reactive astrocytes, antibodies using Axioskope microscope analysis showed NGF in neurons and astrocytes at 28 days after ischaemia (Fig. 5i,l). The majority of the labelled cells were, however, pyramidal neurons in the IE and IC groups, as the area of NGF-MAP-2 merged staining (indicative of neurons; IE: 70 ± 3.2%, IC: 65 ± 0.7%) was larger than that NGF-GFAP merged staining (indicative of reactive astrocytes; IE: 30 ± 3.2%, IC: 35 ± 0.7%) in both groups after 28 days. Figure 4m shows a quantitative comparison of immunolabelled cells in the counted areas of the motor cortex in the IE and IC groups. Two-factor factorial anova revealed significant effects of day (*F*_5,60_ = 23.98, *P*<0.0001), group (*F*_1,60_ = 54.49, *P*=0.003) and the interaction between day and group (*F*_10,60_ = 3.24, *P*=0.01). A subsequent one-way anova (day) revealed a significant difference among groups in number of NGF-labelled cells at 28 days (*F*_1,11_ = 4.84, *P*<0.001) but not at other time points (data not shown). anova analysis demonstrated a significant increase in the number of NGF-labelled cells after exercise (Fig. 5m).

**Figure 6:**
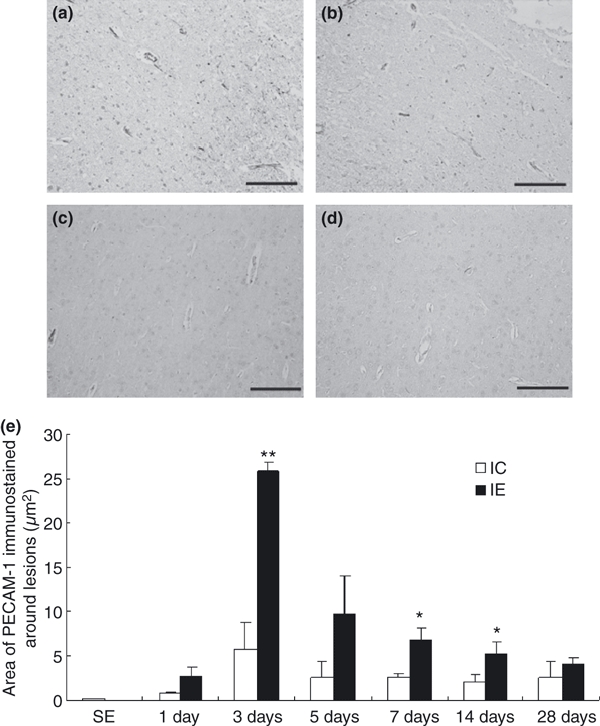
Representative photographs of anti-platelet-endothelial cell adhesion molecule (PECAM-1)-positive cells. Brain sections were immunohistochemically stained for PECAM-1 as an angiogenesis marker in the ipsilateral (a and b) and contralateral (c and d) frontoparietal cortices including the motor cortex 3 days after ischaemic injury in the ischaemia-exercise (IE) (a and c) and ischaemia-non-exercised control (IC) groups (b and d). Changes in the PECAM-1-immunoreactive areas on the infarction side after ischaemia (e) are shown. Quantitative analysis was conducted on PECAM-1 immunoreactive cells from the motor cortex on the infarct side in the IE and IC groups for 28 days after ischaemia that indicated the occurrence of angiogenesis. anova analysis demonstrated a significant increase in the number of PECAM-1 labelled cells after exercise; *P < 0.05 (compared with IC group value), **P < 0.01 (compared with IC group value). Values shown are mean ± SD (*n* = 6). Scale bar = 50 μm.

Platelet-endothelial cell adhesion molecule immunocytochemistry was used to label brain microvessels. PECAM-1-immunopositive cells were consistently detected in the ipsilateral (Fig. 6a,b) and contralateral (Fig. 6c,d) cerebral hemispheres in all groups. In particular, PECAM-1-immunopositive cells were markedly increased in area over a widespread region around the infarct on the ipsilateral side. Quantitative analysis of frontoparietal PECAM-1-immunopositive cells was performed by counting the area of PECAM-1-immunostained microvessels. Two-factor factorial anova revealed significant effects of day (*F*_5,60_ = 53.82, *P*<0.0001), group (*F*_1,60_ = 147.25, *P*<0.0001), and the interaction between day and group (*F*_10,60_ = 26.1, *P*<0.0001). Subsequent one-way anova (day) revealed a significant difference among groups in the area of PECAM-1-immunopositive cells at 3 days (*P*<0.001), 7 days (*P*=0.014) and 14 days (*P*=0.041), but not at other time points (data not shown). In particular, the area of PECAM-1-immunopositive cells was significantly increased in the cells around the infarct in the IE group at 3, 7 and 14 days after surgery (Fig. 6e).

### Cellular expression of caspase-3 after exercise

**Figure 7:**
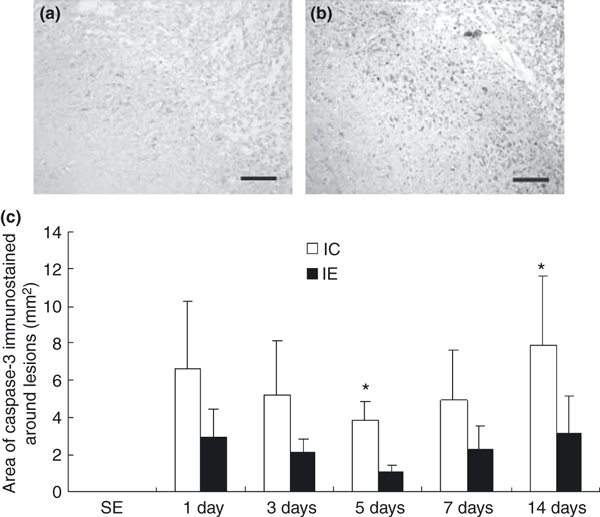
Representative photographs of caspase-3-positive cells. Brain sections were immunohistochemically stained for caspase-3 as an apoptosis marker in the ipsilateral frontoparietal cortex including the motor cortex 14 days after ischaemic injury in the ischaemia-exercise (IE) (a) and ischaemia-non-exercised control (IC) (b) groups. Changes in the caspase-3 -immunoreactive areas on the infarction side after ischaemia (c) are shown. Quantitative analysis was conducted on caspase-3 immunoreactive cells from the motor cortex in the IE and IC groups for 14 days that indicated the occurrence of apoptosis. anova analysis demonstrated a significant increase in the number of caspase-3 labelled cells after exercise; *P < 0.05 (compared with IC group value). No caspase-3 immunoreactive cells were detected in the normal brains (SE group) or at 28 days after ischaemia. Values shown are mean ± SD (*n* = 6). Scale bar = 200 μm.

Photomicrographs of caspase-3-positive cells are shown in Figure 7a,b. Caspase-3-immunopositive cells were detected in the infarct and peri-infarct regions after the induction of ischaemia in rats of the IE and IC groups, but not on the contralateral side nor in the brains of rats of the SE group. The area of caspase-3-positive cells was nearly zero mm^2^ in the SE group brain, but was markedly increased to 7.89 ± 3.68 mm^2^ in the IC group (Fig. 7d) and reduced to 3.29 ± 2.04 mm^2^ in the IE group by 14 days after surgery (Fig. 7c). Two-factor factorial anova revealed a significant effect of group (*F*_1,60_ = 16.38, *P*=0.0003) but no effect of day (*F*_5,60_ = 1.71, *P*=0.17) or of the interaction between group and day (*F*_10,60_ = 0.204, *P*=0.93). A subsequent one-way anova (day) revealed a significant difference among the groups with regard to the number of caspase-3-positive cells at 5 days (*P*<0.01) and 14 days (*P*=0.04), but not at other time points (data not shown). These findings showed that infarction enhanced caspase-3 expression in the ipsilateral frontoparietal cortex including the motor cortex and that treadmill exercise after MCA occlusion significantly suppressed ischaemia-induced increases in caspase-3 expression (Fig. 7c).

## Discussion

Prior to this study, we hypothesized that endogenous growth factors and angiogenesis play roles in the neuroprotective effects of physical exercise against the damage caused by cerebral ischaemia. The present findings suggest that the increases in expression of MK, NGF and PECAM-1 induced by treadmill exercise after ischaemia are strongly related to such neuroprotective effects.

Midkine was expressed in the early stage after the induction of ischaemia, and its expression was significantly increased in the exercised rats in the cells of the peri-infarct region at 3 days after ischaemia. MK was previously found to be expressed in foetal human astrocytes in culture, but not in neurons or oligodendrocytes (Satoh *et al.* 1993). Previous studies identified MK bearing cells as astrocytes in rat experimental cerebral infarction (Yoshida *et al.* 1995, Wang *et al.* 1998), and spinal cord injury (Sakakima *et al.* 2004a,b;) with double immunostaining using rabbit anti-MK and mouse anti-GFAP antibodies. The expression of GFAP, a marker of reactive astrocytes, was found in the zones surrounding infarcts. MK is produced around the lesion and may function as a reparative neurotrophic factor during the early phase after cerebral infarction (Yoshida *et al.* 1995). Moreover, MK delayed the process of neuronal death after forebrain ischaemia in gerbils (Yoshida *et al.* 2001) and neuronal death upon ischaemic brain injury was partly prevented by the introduction of the MK gene (Takada *et al.* 2005). MK promotes neuronal survival and plays important roles in the development and preservation of inflammation as well as in the repair of injured tissues (Friedrich *et al.* 2005, Horiba *et al.* 2006, Sakakima *et al.* 2006, Zou *et al.* 2006). However, the promotion of MK expression by treadmill exercise was recognized in this experiment, and its role remains to be clarified.

We detected expression of PECAM-1, a marker of angiogenesis, and caspase-3, a marker of apoptosis, at later time points compared to MK expression. MK-, PECAM-1- and caspase-3-immunopositive cells were observed in the peri-infarct region from relatively early after infarction (1–5 days for MK, 3–14 days for PECAM-1 and 5–14 days for caspase-3) and were increased or decreased in number in the same area of the brains of the exercised rats. These findings suggest that neurotrophic factors such as MK are expressed after brain injury and protect neurons around infarcts from injury. Physical activity on a running wheel increases blood vessel density in the brain (Kleim *et al.* 2002), and daily forced exercise on a treadmill induces cortical and striatal angiogenesis in rats (Ding *et al.* 2004a,b;). Moreover, wheel running for 3 weeks reduced the expression of mRNA for the apoptosis-associated genes Bcl-x and neuronal death protein (DP5) (Tong *et al.* 2001), and treadmill exercise was shown to decrease lesion size and suppress the enhancement of caspase-3 expression following intrastriatal haemorrhage (Lee *et al.* 2003a,b;). The reduction in infarct volume in the IE group relative to that of the ischaemia-non-exercised control group and the promotion of MK and PECAM-1-positive cells and the inhibition of caspase-3-positive cells may be associated or may be dependent phenomena. The present study does not reveal a direct association, but rather provides indirect evidence of such an association.

In addition, NGF is of particular interest with regard to the promotion of cell growth. The expression of NGF significantly increases at 6 h post-ischaemia and then quickly returns to normal levels over 12 h to 7 days in rats with cerebral infarction (Chang *et al.* 2005). However, a recent study suggested that the neuroprotective effect associated with 12 weeks of physical exercise before permanent MCA occlusion was due to an increase in endogenous NGF (Ang *et al.* 2003). In the present study, the level of NGF protein detected by immunostaining was significantly higher in the IE group than in the IC group at 28 days (*P* < 0.05). Exercise-induced NGF expression could therefore play a role in reducing brain injury around 4 weeks after ischaemic stroke. Exercising animals with stroke-induced brain injury increases the expression of other neurotrophic factors, such as BDNF, NGF, HGF, HIF-1, and bFGF, which regulate neuronal survival and differentiation as well as synaptic plasticity in the hippocampus and cerebral cortex (Gonez-Pinilla *et al.* 1998, Ang *et al.* 2003, Ding *et al.*, 2004b; Endres *et al.* 2003, Kim *et al.* 2005). Although numerous neuroprotective mechanisms probably occur following treadmill exercise, the increase in neurotrophic expression of NGF found in the present study may be the result of heightened neuronal activity during exercise.

Most research has focused on the acute stage of cerebral ischaemia and has included investigation of neither the progression of changes in the brain nor late changes. In the present study, we examined changes from the acute to the subacute stage of cerebral ischaemia and found that the expression of MK was upregulated early after ischaemia while that of NGF was upregulated later after cerebral ischaemia. These findings suggest that neurotrophic factors play roles in neuronal survival and proliferation and exhibit neuroprotective functions in different phases after the induction of ischaemia. Further studies are needed to clarify the effects of increases in the levels of neurotrophic factors such as MK and NGF in the various phases following ischaemia on survival.

Physical activity can induce neuroplastic adaptations and improve outcomes after cerebral injury (Marin *et al.* 2003). Some experimental findings have shown that general activation starting 24 h after an ischaemic event promotes functional outcome without increasing tissue loss (Ohlsson & Johansson 1995, Johansson & Ohlsson 1996). However, forced overuse of impaired forelimbs during the first 7 days after brain injury results in the expansion of the neural injury in the areas representing the forelimb in the sensorimotor cortex in rats and strongly interferes with the restoration of function (Humm *et al.* 1998). Constraint-induced movement therapy immediately after ischaemic brain injury was found to worsen brain injury and delay functional recovery (Bland *et al.* 2000). Early intense and stressful training involving forced arm use has also been found to interfere with recovery (Kozlowski *et al.* 1996, Humm *et al.* 1998).

On the other hand, environmental enrichment significantly improves functional outcome in ischaemic rats. However, wheel running is ineffective at improving motor function (Resedal *et al.* 2002). Exercise on a treadmill, but not wheel exercise or restraint-induced movement therapy, reduced infarct volume (Hayes *et al.* 2008). Controversy continues to exist regarding the effects of exercise on brain ischaemia. The intensity of exercise is an important factor in this. In this study, the rats underwent treadmill running at a speed of 3–13 m min^−1^ for 20 min a day every day for a maximum of 28 days. The intensity of treadmill exercise training at 16–20 m min^−1^ was approx. 55%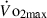 in hypertensive rats (Véras-Silva *et al.* 1997). Moreover, running at a speed of 8–20 m min^−1^ was found to enhance functional recovery after ischaemia, and early appropriate exercise improved recovery from brain ischaemia (Wang *et al.* 2001, Lee *et al.* 2003a,b;, Yang *et al.* 2003, Ding *et al.* 2004a,b;). The progressive treadmill exercise provided in this study was sufficient to improve motor function and neurological deficits.

One limitation of this study is that we used a rat stroke model to simulate human stroke. Although MCA infarction is the most common type of stroke in humans, infarctions do occur in other brain regions. In addition, we used only immunochemical techniques to detect the expression of midkine, NGF, PECAM-1 and caspase-3; therefore, this study was deficient in stereological analysis. We tried Western blot analysis but failed to detect MK protein because of the very small amounts present in the brain, compared to those of the other proteins. Although further studies are needed to provide direct evidence showing the causal relationship between exercise-induced motor improvement and cellular expression of neurotrophins or angiogenesis, the present study is suggestive of the contribution of the ameliorative effect of treadmill exercise on cerebral infarction.

In conclusion, treadmill exercise in rats subjected to transient MCA occlusion was found to improve motor behaviour and reduce neurological deficit and infarct volume. Cellular expression of MK and NGF, and angiogenesis were significantly increased in the cells around the infarctions of the rats undergoing exercise. In addition, treadmill exercise was shown to suppress caspase-3 expression around the site of ischaemia. Early, low-intensity rehabilitation exercise thus appears to aid recovery from brain damage and functional outcome after stroke.
